# Acetylsalicylic acid use is associated with reduced risk of out-of-hospital cardiac arrest in the general population: Real-world data from a population-based study

**DOI:** 10.1371/journal.pone.0267016

**Published:** 2022-06-08

**Authors:** Talip E. Eroglu, Marieke T. Blom, Patrick C. Souverein, Alfi Yasmina, Anthonius de Boer, Hanno L. Tan, for the ESCAPE-NET investigators

**Affiliations:** 1 Department of Cardiology, Heart Center, Academic Medical Center, University of Amsterdam, Amsterdam, The Netherlands; 2 Division of Pharmacoepidemiology and Clinical Pharmacology, Utrecht Institute for Pharmaceutical Sciences, Utrecht University, Utrecht, The Netherlands; 3 Department of Cardiology, Copenhagen University Hospital–Herlev and Gentofte, Copenhagen, Denmark; 4 Department of Pharmacology, Faculty of Medicine, Lambung Mangkurat University, Banjarmasin, Indonesia; 5 Netherlands Heart Institute, Utrecht, The Netherlands; University of Tampere, FINLAND

## Abstract

**Aim:**

Activated blood platelet products facilitate myocardial intracellular Ca^2+^ overload, thereby provoking afterdepolarizations and increasing susceptibility of ischemic myocardium to ventricular fibrillation (VF). These effects are counteracted *in vitro* by acetylsalicylic acid (ASA), but no prior study investigated whether ASA is associated with decreased out-of-hospital cardiac arrest (OHCA) risk on a population level. Therefore, we studied whether ASA and other antiplatelet drugs (carbasalate calcium, clopidogrel) are associated with decreased risk of OHCA.

**Methods:**

We conducted a population-based case-control study among individuals (772 OHCA-cases with documented VT/VF, 2444 non-OHCA-controls) who had used antiplatelet drugs in the year before index-date (OHCA-date), and studied the association between current antiplatelet drug use and OHCA-risk with multivariable logistic regression analysis.

**Results:**

ASA use was associated with reduced OHCA-risk (adjusted odds ratio (OR_adj_) 0.6 [0.5–0.8]), and more so in women (OR_adj_ 0.3 [0.2–0.6]) than in men (OR_adj_ 0.7 [0.5–0.95], P_interaction_ 0.021). Carbasalate calcium was associated with decreased OHCA-risk in women (OR_adj_ 0.5 [0.3–0.9]), but not in men (OR_adj_ 1.3 [0.96–1.7], P_interaction_ 0.005). Clopidogrel was not associated with reduction in OHCA-risk. Risk reduction associated with ASA in patients with OHCA was similar in the presence of acute myocardial infarction (AMI) (OR_adj_ 0.6 [0.4–0.9]) and in the absence of AMI (OR_adj_ 0.7 [0.4–1.2]).

**Conclusion:**

ASA use was associated with reduced OHCA-risk in both sexes, and more so in women, while carbasalate calcium only protected women. Clopidogrel was not associated with reduced OHCA-risk.

## Introduction

Out-of-hospital cardiac arrest (OHCA) accounts for 50% of all deaths from cardiovascular disease [1], and is mostly caused by cardiac arrhythmia (ventricular tachycardia/ventricular fibrillation [VT/VF]), often triggered by acute myocardial infarction (AMI) [2]. Previously, we reported that substances released from activated blood platelets (activated blood platelet products [ABPPs]) impact cardiac electrophysiological properties. In particular, ABPPs facilitate intracellular Ca^2+^ overload by increasing intracellular calcium transients and L-type calcium current (without affecting sarcolemmal sodium or potassium currents) [3, 4]. These changes may increase susceptibility of ischemic myocardium to VF by provoking early afterdepolarizations (EADs) or delayed afterdepolarizations (DADs), but they were counteracted *in vitro* by acetylsalicylic acid (ASA) [4]. ASA derives its therapeutic action from inhibition of cyclooxygenase; this prevents the formation of cyclooxygenase products, such as thromboxane and prostaglandins (two types of ABPP). Given these observations, it is possible that ASA reduces the risk of VT/VF and OHCA. Women, in particular, may benefit from these antifibrillatory actions of ASA and, possibly, other antiplatelet drugs (carbasalate calcium, clopidogrel), because they are more vulnerable than men to the occurrence of EADs and DADs given their higher expression levels of cardiac L-type calcium currents [5]. However, it has not been studied yet whether or not ASA, carbasalate calcium or clopidogrel lower the risk for OHCA on a population level; to establish this was the aim of our present investigation. To do so, we performed a case-control study using data from population-based registries in the Netherlands. Because we expected that women, in particular, may benefit from the antifibrillatory actions of antiplatelet drugs, we stratified our analysis according to sex. Furthermore, expecting that OHCA risk reduction primarily occurs when ABPPs are formed, we studied the possible differential effects of antiplatelets in the presence or absence of ABPP formation. Taking acute myocardial infarction (AMI) as a situation in which ABPP formation is present, we performed subgroup analysis of OHCA cases who suffered OHCA in the presence or absence of AMI.

## Methods

### Design and setting

We performed a population-based case-control study. Cases were OHCA victims from cardiac causes with ECG-documented VT/VF enrolled in the AmsteRdam REsuscitation STudy (ARREST [6]) registry in 2005–2011. We excluded cases without complete drug-dispensing records one year prior to index-date (OHCA date), those with obvious non-cardiac causes (e.g., trauma, drowning), and those who suffered their second or subsequent OHCA episode. Controls were individuals who did not have OHCA at or before the index-date. For each case, up to five controls who were alive on the index-date were matched using exact matching based on age, sex, and index-date. From this original case-control data set, individuals aged ≥18 years with coronary artery disease, defined by use of an antiplatelet drug in the 12 months before index-date, were included in the present analysis. Thus, the majority of cases and controls were treated for secondary prevention of cardiovascular disease, thereby increasing comparability with respect to underlying conditions. By sub-selecting all individuals who had used an antiplatelet drug in the year before the index-date from the original case-control data set, the original matching was lost.This study was conducted based on the principles outlined in the Declaration of Helsinki, and was approved by the Medical Ethics Committee of the Academic Medical Center, Amsterdam.

### Data sources

Details of the ARREST registry are described elsewhere [6]. In brief, ARREST is an ongoing population-based registry that prospectively enrolls all suspected OHCAs in one contiguous region of the Netherlands (North Holland province, covering both urban and rural areas and containing approximately 2.4 million individuals) in collaboration with all dispatch centers, Emergency Medical Services (EMS) and hospitals in the study region. OHCA was defined as an EMS-attended resuscitation attempt for a cardiac arrest with presumed cardiac cause. After each suspected OHCA, EMS routinely provides the continuous ECG from their manual defibrillators. If an automated external defibrillator is used prior to EMS arrival, the ECG from the automated external defibrillator is obtained by the ARREST personnel. The presence or absence of VT/VF was verified from these ECGs [6]. Information regarding the immediate cause of VT/VF was obtained from hospital records and could only be obtained from patients who survived to hospital admission. The immediate cause of VT/VF was classified as AMI or no AMI (any other cardiac cause), or unknown. The diagnosis of AMI was established by the treating cardiologist and retrieved retrospectively from hospital records [6]. Information regarding drug-dispensing records one year prior to index-date was obtained by contacting the patient’s community pharmacy using standardized protocols. Controls were sampled from the PHARMO Database Network that contains, among other things, drug-dispensing records from community pharmacies [7]. We also obtained drug dispensing records one year prior to index-date from controls. As virtually all individuals in the Netherlands are registered at a single pharmacy, drug-dispensing records are considered as complete [8].

### Exposure of interest and covariates

Current use of antiplatelet drugs was defined as having a drug-dispensing record within 90 days prior to index-date since, in the Netherlands, the average repeat prescription length for drugs used for chronic diseases is 90 days. We used the Anatomical Therapeutical Chemical (ATC) Classification System to define the use of antiplatelet drugs (B01AC). In order to estimate OHCA risk upon use of individual antiplatelet drugs, we included the most commonly used antiplatelet drugs in the Netherlands, i.e., ASA (B01AC06), carbasalate calcium (B01AC08), and clopidogrel (B01AC04). The association between other antiplatelet drugs (dipyridamole, ticlopidine) was not evaluated because the number of users was too small to yield meaningful results. High dose ASA used as analgesic has a different ATC code (N02BA01) than as antiplatelet drug (B01AC06) and was not included in this study. The association between individual antiplatelet drugs and OHCA risk could not be estimated in case of combinations of individual antiplatelet drugs, therefore these ORs are not reported.

### Covariates

As covariates, we considered age, sex and pre-existing disease with known effects on OHCA risk (cardiovascular disease, diabetes mellitus) by using drug proxies (cardiovascular drugs and antidiabetics, respectively, both defined as use within six months before index-date, listed in Table 1). We could not obtain diagnoses regarding pre-existing disease in the control group, but used drug use as proxies, as we did previously [9, 10]. Drugs used to define pre-existing disease are usually taken chronically. Also, medications with known effects on OHCA were evaluated: non-cardiac QT-prolonging drugs (from www.CredibleMeds.org [11, 12]) and antiarrhythmic drugs (Vaughan-Williams class 1 or 3). Use of non-cardiac QT-prolonging drugs and/or Vaughan-Williams class 1 or 3 antiarrhythmic drugs was defined as use within 90 days before index-date.

**Table 1:** Baseline characteristics of cases and controls.

	Cases	Controls	p-value
Total	772	2444	
Mean age, years (SD)	70.4 (11.5)	73.1 (10.1)	<0.001
Male sex	623 (80.7)	2033 (83.2)	0.113
Drugs used in the 6 months before index date			
Beta-blockers	443 (57.4)	1152 (47.1)	<0.001
Calcium channel blockers	203 (26.3)	614 (25.1)	0.514
Renin angiotensin system inhibitors	477 (61.8)	1151 (47.1)	<0.001
Diuretics	382 (49.5)	838 (34.3)	<0.001
Nitrates	241 (31.2)	441 (18.0)	<0.001
Statins	497 (64.4)	1456 (59.6)	0.017
Antidiabetic drugs	193 (25.0)	480 (19.6)	0.001
Antiarrhythmic drugs class 1 or 3	17 (2.2)	12 (0.5)	<0.001
Non-antiarrhythmic QT-prolonging drugs	48 (6.2)	88 (3.6)	0.002

Numbers are number (%) unless indicated otherwise

### Statistical analysis

The association between current antiplatelet drug use and OHCA risk was assessed by employing multivariable logistic regression analyses using 2 models. In model 1, estimates were adjusted for age and sex, and in model 2, estimates were additionally adjusted for use of cardiovascular drugs, antidiabetics, non-cardiac QT-prolonging drugs and/or antiarrhythmic drugs (listed in Table 1). By sub-selecting the original case-control data set to individuals who had used an antiplatelet drug in the year before the index-date, matching was lost. Therefore, we adjusted for age and sex in all analyses. First, we studied the association between current use of any antiplatelet drug and OHCA risk versus no current use of any antiplatelet drug. Second, we studied the association between current use of individual antiplatelet drugs (ASA, carbasalate calcium, clopidogrel) and OHCA risk compared to no current use of any antiplatelet drug in the whole group, and stratified according to sex. Third, we assessed risk of current use of antiplatelet drugs in the subgroups of OHCA cases who suffered OHCA in the presence or absence of AMI (compared to all controls). The presence of interaction on a multiplicative scale between sex and antiplatelet drugs was estimated by including the cross-product of the two factors as a variable in the model. Results are presented as odds ratio (OR) and 95% confidence interval (CI). Descriptive statistics are reported as mean (standard deviation) or number (percent) as indicated. Comparisons for continuous variables were made with independent t-test. Chi-square test was used when discrete variables were compared across groups. A two-sided p-value of <0.05 was considered statistically significant.

## Results

**Fig 1:**
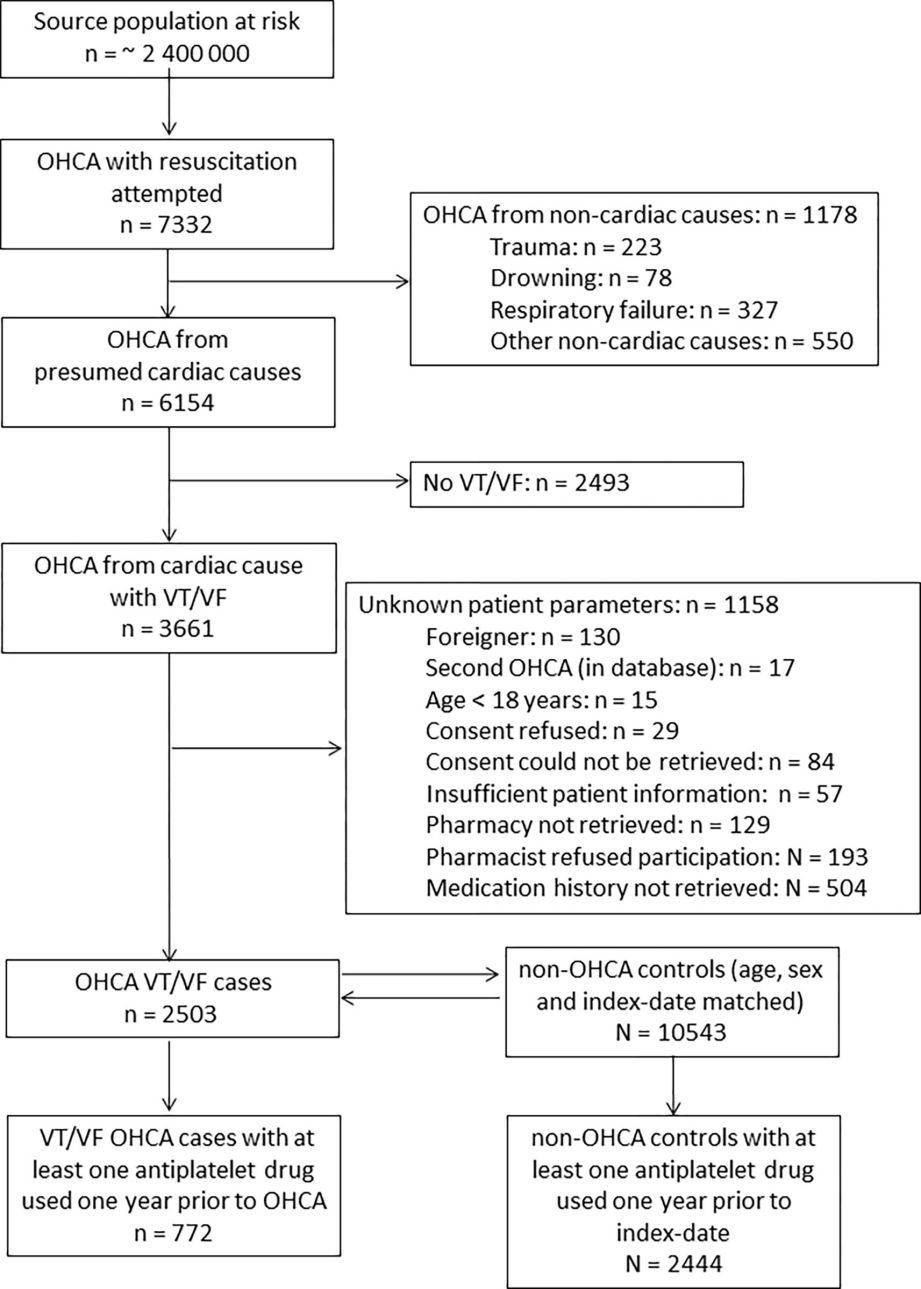
Flow chart of inclusion of out-of-hospital cardiac arrest cases. Abbreviations: OHCA, out-of-hospital cardiac arrest; VT/VF, ventricular tachycardia/ventricular fibrillation.

We identified 2503 OHCA-cases with cardiac causes, ECG-documented VT/VF, and complete drug-dispensing records; among these cases, 772 (mean age 70.4 years, 80.7% male, Table 1) used one or more antiplatelet drug in the year before OHCA-date (Fig 1). Among 10,543 non-OHCA controls with complete drug-dispensing records, 2444 (mean age 73.1 years, 83.2% male, Table 1) used one or more antiplatelet drug in the year before index-date (Fig 1).

**Fig 2:**
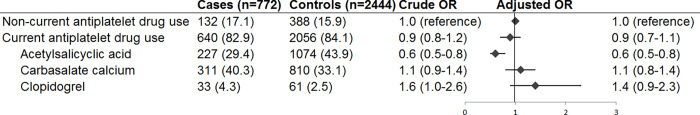
Current use of antiplatelet drugs, acetylsalicylic acid, carbasalate calcium or clopidogrel and out-of-hospital cardiac arrest risk compared with no current use of any antiplatelet drugs. Not included in the figure: current users of other antiplatelet monotherapy (e.g., dipyridamole, ticlopidine) among cases (n = 9, 1.2%) and controls (n = 42, 1.7%), and current users of ≥2 antiplatelet drugs among cases (n = 60, 7.8%) and controls (n = 69, 2.8%). Abbreviations: CI, confidence interval; OR, odds ratio. Numbers in table are number (%) unless indicated otherwise. Error bars denote 95% confidence interval. Numbers are number (%) unless indicated otherwise.

**Fig 3:**
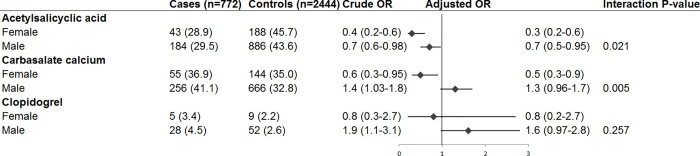
Current use of acetylsalicylic acid, carbasalate calcium or clopidogrel and out-of-hospital cardiac arrest risk compared with no current use of any antiplatelet drugs: Stratified according to sex. Not included in the figure: current users of other antiplatelet monotherapy (e.g., dipyridamole, ticlopidine) among cases and controls, and current users of ≥2 antiplatelet drugs among cases and controls. Abbreviations: CI, confidence interval; OR, odds ratio. Numbers in table are number (%) unless indicated otherwise. Error bars denote 95% confidence interval. Numbers are number (%) unless indicated otherwise.

**Fig 4:**
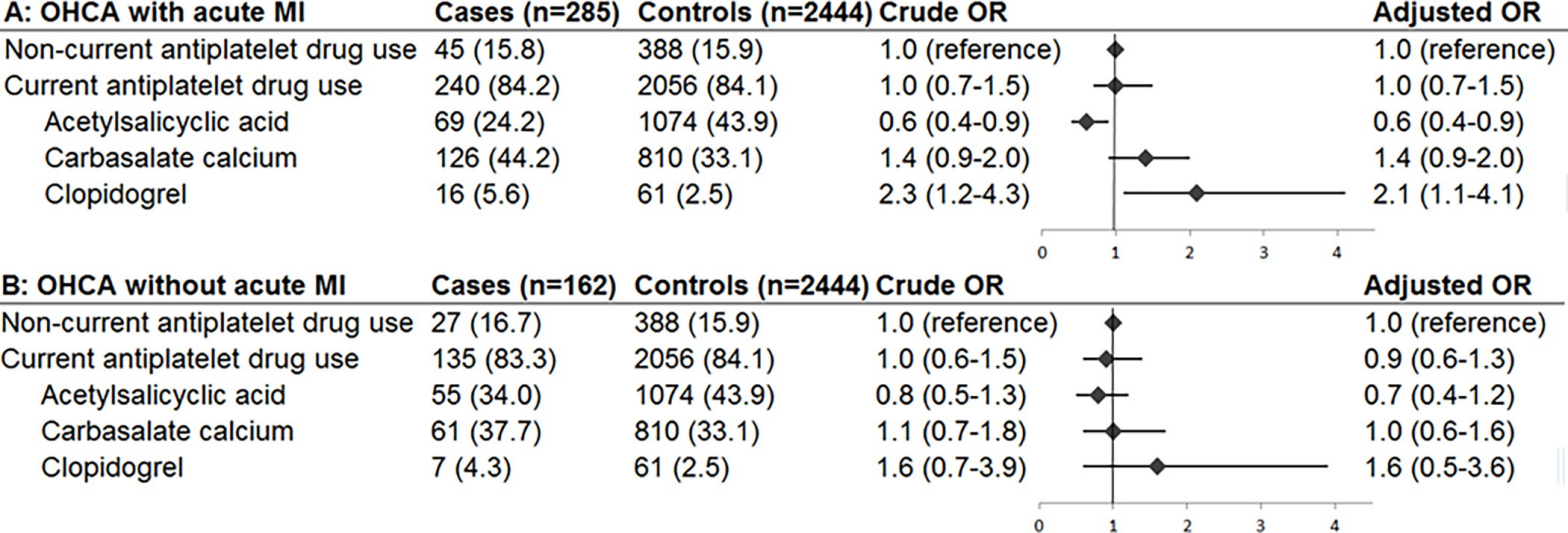
Current use of antiplatelet drugs and the risk of out-of-hospital cardiac arrest with acute myocardial infarction as the cause (A) and without acute myocardial infarction as the cause (B). Not included in the figure: current users of other antiplatelet monotherapy (e.g., dipyridamole, ticlopidine) among cases and controls, and current users of ≥2 antiplatelet drugs among cases and controls. Abbreviations: CI, confidence interval; MI, myocardial infarction; OHCA, out-of-hospital cardiac arrest; OR, odds ratio. Numbers in table are number (%) unless indicated otherwise. Error bars denote 95% confidence interval.

When antiplatelet drugs were studied as a group, current use of any antiplatelet drug was not associated with OHCA risk (OR_adj_ 0.9 [95% CI 0.7–1.1], Fig 2). When we studied individual drugs, we found that current ASA use was associated with decreased OHCA risk (OR_adj_ 0.6 [95% CI 0.5–0.8). This risk reduction was larger in women (OR_adj_ 0.3 [95% CI 0.2–0.6]) than in men (OR_adj_ 0.7 [95% CI 0.5–0.95], P_interaction_ 0.021, Fig 3). Current carbasalate calcium use was associated with decreased OHCA risk in women (OR_adj_ 0.5 [95% CI 0.3–0.9]), but not in men (OR_adj_ 1.3 [95% CI 0.96–1.7], P_interaction_ 0.005, Fig 3). Current clopidogrel use was not associated with reduced OHCA risk in the overall model with both sexes (OR_adj_ 1.4 [CI 0.9–2.3], Fig 2). The risk reduction associated with current ASA use in patients who suffered OHCA following AMI (OR_adj_ 0.6 [0.4–0.9], Fig 4) was similar as in patients who suffered OHCA without AMI (OR_adj_ 0.7 [0.4–1.2], Fig 4).

To examine why reduced OHCA risk was observed among current ASA users (both sexes), but not among carbasalate calcium users, we assessed whether concomitant medication use was different between these groups. We found no such differences between ASA users and carbasalate calcium users (Table 2).

**Table 2:** Characteristics of clopidogrel, acetylsalicylic acid and carbasalate calcium users.

	clopidogrel	acetylsalicylic acid	carbasalate calcium	p-value
Total	94	1301	1121	
Mean age, years (SD)	71.3 (10.4)	73.2 (10.2)	72.1 (10.4)	0.009
Male sex	80 (85.1)	1070 (82.2)	922 (82.2)	0.775
Drugs used in the 6 months before index date				
Beta-blockers	55 (58.5)	655 (50.3)	551 (49.2)	0.213
Calcium channel blockers	31 (33.0)	340 (26.1)	262 (23.4)	0.060
Renin angiotensin system inhibitors	53 (56.4)	661 (50.8)	580 (51.7)	0.558
Diuretics	41 (43.6)	484 (37.2)	430 (38.4)	0.434
Nitrates	26 (27.7)	241 (18.5)	237 (21.1)	0.047
Statins	62 (66.0)	836 (64.3)	677 (60.4)	0.116
Antidiabetic drugs	20 (21.3)	274 (21.1)	245 (21.9)	0.893
Antiarrhythmic drugs class 1 or 3	0	7 (0.5)	11 (1.0)	0.306
Non-antiarrhythmic QT-prolonging drugs	5 (5.3)	46 (3.5)	56 (5.0)	0.181

Numbers are number (%) unless indicated otherwise

## Discussion

In this observational study using real-world population-based data, we found that current ASA use was associated with reduced risk of OHCA. This protective effect was larger in women than in men. Carbasalate calcium use only reduced OHCA risk in women, but not in men. Clopidogrel use was not associated with reduced OHCA risk in either sex. Risk reduction by ASA among patients who suffered OHCA following AMI was similar to risk reduction among patients without AMI.

Previously, we demonstrated sex disparities in susceptibility to EADs by showing that ventricular myocytes of women are more susceptible to EADs than those of men. This could be explained by larger depolarizing L-type Ca^2+^ current in conjunction with smaller transient outward potassium current in women than in men [13]; these observations were consistent with animal studies [14]. Given the importance of L-type Ca^2+^ current during the action potential plateau, even mild increases in this current could lead to longer action potentials in women, rendering them more vulnerable to EADs than men. Accordingly, women may benefit more than men from the blocking effects of ASA and carbasalate calcium on ABPP-induced increase in L-type Ca^2+^ current and intracellular Ca^2+^ transients, explaining our observation that ASA and carbasalate calcium are associated with more OHCA risk reduction in women than in men.

Although these mechanisms might explain lower OHCA risk among ASA users, they do not explain why carbasalate calcium was associated with less reduction in OHCA risk than ASA in the overall model with both sexes (Fig 2). Carbasalate calcium is a calcium salt of ASA and is quickly metabolized to ASA after absorption [15]. Both drugs inhibit the conversion of arachidonic acid to prostaglandins and thromboxane by inhibiting cyclooxygenase. Given that we found no indications that the differences in observed effects between ASA and carbasalate calcium could be explained by specific drug properties, we studied whether patient differences may be responsible. When we studied concomitant medication use, we found no statistically significant differences in concomitant medication use between ASA users and carbasalate calcium users, nor between men who used ASA or carbasalate calcium (S1 Table). We may speculate that residual confounders may play a role, since we lack important data on risk factors for OHCA such as left ventricular ejection fraction, smoking, BMI. Moreover, we may speculate that it could be that there may be a difference in the therapeutic indication for which the antiplatelet drugs were prescribed between subgroups (coronary artery disease, cerebrovascular events or peripheral artery disease), which may have affected the association with OHCA. However, we could not test this, since we have no information regarding therapeutic indications. In any case, carbasalate calcium is currently not recommended by the guidelines [16]. Consequently, the use of carbasalate calcium has declined by almost a half in the Netherlands between 2014 and 2018 [17]. Taken together, our results may support the use of ASA above carbasalate calcium when platelet inhibition is indicated.

Our findings are consistent with animal studies which showed that thrombotic coronary occlusion caused greater incidence of VT/VF than non-thrombotic occlusion, despite similar infarct sizes [18, 19], thereby suggesting that the intracoronary thrombus itself increases the vulnerability of ischemic myocardium to VT/VF above and beyond the effects of acute ischemia [18]. Supporting this hypothesis, various animal studies were conducted to examine the effects of ABPPs on cardiac electrophysiology [3, 20]. Increases of the inward L-type Ca^2+^ current and intracellular Ca^2+^ transients in cardiac myocytes were found after exposure of rabbit ventricular myocytes to ABPPs; this resulted in prolongation of action potential duration and the occurrence of EADs and DADs [3]. Moreover, platelets release various arrhythmogenic compounds during ischemia, such as thromboxane, which facilitate VF independently of their ability to participate in the formation of an occlusive thrombus [20]. Accordingly, we provided evidence in a previous study that cyclooxygenase products of arachidonic acid, such as thromboxane and prostaglandins, are responsible for the increase in inward L-type Ca^2+^ current and intracellular Ca^2+^ transients in cardiac myocytes [4] by showing that ASA pretreatment significantly reduced the effects of ABPP on cellular calcium homeostasis [4]. Moreover, a previous study showed that all of the prostaglandins (i.e. PGD_2_, PGE_2_, PGF_2α_) derived from arachidonic acid are arrhythmogenic, of which PGF_2α_ is the most potent to induce tachyarrhythmias in cultured neonatal rat myocytes [21]. Thus, reducing the effects of ABPP on inward L-type Ca^2+^ current and intracellular Ca^2+^ transients may contribute to the decrease in OHCA risk by ASA. This may be a mechanism underlying our observation that ASA reduces OHCA risk. Previous studies found antiarrhythmic effects of treatment with ASA during ischemia in dogs [22], while intravenous administration of ASA reduced ischemia induced VF in rats [23]. This is in accordance with our findings which demonstrated reduced OHCA risk in the setting of AMI upon ASA use. Moreover, a study among patients with acute coronary syndrome found that patients who suffered pre-hospital cardiac arrest (without VT/VF documentation) were less frequently treated with ASA than patients without pre-hospital cardiac arrest [24]. However, the exposure window of ASA in that study was defined as the use of ASA prior to hospital presentation, which carries the risk of misclassification of ASA use by including past users (rather than current users) in the analysis.

Based on the described mechanism of platelet-derived products from the cyclooxygenase pathway and their relation with VF occurrence, it is expected that clopidogrel is not associated with lower OHCA risk [25–28]. ASA and clopidogrel act by different mechanisms. Clopidogrel attenuates the secondary response to adenosine diphosphate by blocking the P2Y_12_ receptor, thereby inhibiting thrombus formation, but not the production of thromboxane A2 and prostaglandins, two type of ABPPs that may elicit proarrythmic effects *in vitro* [3]. In contrast, ASA does inhibit the production of both compounds directly by inhibiting cyclooxygenase 1 [25–28]. Our findings were consistent with this expectation, and supported by an experimental study which showed that clopidogrel pre-treatment did not reduce ischemia-induced VF incidence [20], and a clinical report that, among patients with acute coronary syndrome, clopidogrel use was not different between those with or without pre-hospital cardiac arrest [24]. We observed an increased risk of OHCA in the setting of AMI upon use of clopidogrel. However, clopidogrel is usually given as secondary prevention to patients with acute coronary syndrome or percutaneous coronary intervention and after intracoronary stent implantation, and we acknowledge that the possibility of confounding by indication cannot be ruled out. Future studies are required to establish the molecular compounds responsible for the proarrhythmic effects of ABPPs; these studies may identify new targets for drug development for prevention of ischemia induced VF.

### Strengths and limitations

A major strength of ARREST registry is the population-based real-world design in which every OHCA was obtained prospectively from the general population; this reduces the risk for selection bias. Furthermore, by studying the general population, including both urban and rural areas and capturing >90% of all OHCAs, our findings are representative for the community at large. Furthermore, to ascertain that OHCA resulted from cardiac causes, ECGs were obtained, and all OHCAs from obvious non-cardiac causes were excluded. Another strength is that information regarding drug use was based on drug-dispensing records in both cases and controls, an important step closer to the actual drug intake than only drug prescription records. Furthermore, we have no reason to assume that actual drug intake would be different between cases and controls, or between users of different antiplatelet drugs. Hence, any possible misclassification regarding drug intake is expected to be similarly distributed between cases and controls (non-differential misclassification). A limitation is that we had no information on relevant comorbidities for the majority of our cases and in all for the controls. Therefore, we could not perform direct adjustments for relevant comorbidities. To deal with this, we used as a proxy for disease drug-dispensing records obtained from community pharmacies, considered a reliable source for drug exposure [29]. Another limitation associated with the lack of data on comorbidities is that we had no information on therapeutic indication. Considering that antiplatelet drugs have a different range of indications (e.g., coronary artery disease, cerebrovascular events or peripheral artery disease), the underlying disease for which they are prescribed may have affected the association with OHCA. Secondly, confounding by indication might play a role in our study. To overcome this, we selected all patients who had at least one antiplatelet drug prescription prior to index-date, so most of the patients were treated for secondary prevention of cardiovascular disease. However, it is still possible that (unmeasured) residual confounders might have affected our results, since data on several important risk factors such as left ventricular ejection fraction and systolic function were not available. Moreover, given the highly unpredictable way in which OHCA occurs, it is difficult, if not impossible, to obtain such data shortly before OHCA occurrence in a uniform manner across the study population. Another limitation is that we could only obtain AMI status for OHCA cases who survived to hospital admission. In OHCA cases who died before hospital admission, a diagnosis could not be made; these cases were therefore excluded from our subgroup analyses according to AMI status. We used documented AMI as a specific marker of ischemia during OHCA. Yet, the sensitivity of this marker may be low, and in some patients OHCA may have occurred during an ischemia episode that did not lead to AMI, e.g., because of spontaneous reperfusion by thrombus resolution and/or relaxation of the culprit vessel [30]. This might explain why we did not observe different associations in the AMI and non-AMI groups. Finally, the observational nature of our study allows for reporting statistical associations, and, as such, we could only detect associations without proving causality. Furthermore, our subgroup analysis was based on small sample sizes, which may have resulted in possible low statistical power. Hence, our findings should be interpreted with caution. Future studies with available data on therapeutic indication are needed to confirm and support our findings.

## Conclusion

ASA use was associated with decreased risk of OHCA in both sexes, but was larger in women than in men, while carbasalate calcium use only reduced OHCA risk in women, but not in men. Clopidogrel use was not associated with reduction in OHCA risk.

## Supporting information

S1 Table Characteristics of males that used acetylsalicylic acid or carbasalate calcium. (DOCX)
