# Supplementary material for: Acetylsalicylic acid use is associated with reduced risk of out-of-hospital cardiac arrest in the general population: Real-world data from a population-based study
Source: PLoS One. 2022 Jun 8;17(6):e0267016. doi: 10.1371/journal.pone.0267016 (PMC9176768; doi:10.1371/journal.pone.0267016)
Supplement: S1 Table — (DOCX) [file pone.0267016.s001.docx]

| **Supplemental Table 1.** Characteristics of males that used acetylsalicylic acid or carbasalate calcium | | | |
| --- | --- | --- | --- |
|  | acetylsalicylic acid | carbasalate calcium | p-value |
| Total | 1070 | 922 |  |
| Mean age, years (SD) | 72.3 (10.1) | 71.5 (10.4) | 0.057 |
| Drugs used in the 6 months before index date |  |  |  |
| Beta-blockers | 542 (50.7) | 460 (49.9) | 0.734 |
| Calcium channel blockers | 280 (26.2) | 214 (23.2) | 0.127 |
| Renin angiotensin system inhibitors | 547 (51.1) | 470 (51.0) | 0.948 |
| Diuretics | 369 (34.5) | 330 (35.8) | 0.543 |
| Nitrates | 192 (17.9) | 195 (21.1) | 0.071 |
| Statins | 706 (66.0) | 570 (61.8) | 0.054 |
| Antidiabetic drugs | 213 (19.9) | 208 (22.6) | 0.148 |
| Antiarrhythmic drugs class 1 or 3 | 5 (0.5) | 8 (0.9) | 0.268 |
| Non-antiarrhythmic QT-prolonging drugs | 39 (3.6) | 45 (4.9) | 0.171 |
| Numbers are number (%) unless indicated otherwise | | | |
